# Altered explorative strategies and reactive coping style in the FSL rat model of depression

**DOI:** 10.3389/fnbeh.2015.00089

**Published:** 2015-04-21

**Authors:** Salvatore Magara, Sarah Holst, Stina Lundberg, Erika Roman, Maria Lindskog

**Affiliations:** ^1^Department of Neuroscience, Karolinska InstitutetStockholm, Sweden; ^2^Department of Pharmaceutical Biosciences, Uppsala UniversityUppsala, Sweden

**Keywords:** multivariate concentric square field (MCSF) test, novel cage test, home cage change test, risk assessment, risk taking, social behavior, behavioral profiling, coping style

## Abstract

Modeling depression in animals is based on the observation of behaviors interpreted as analog to human symptoms. Typical tests used in experimental depression research are designed to evoke an either-or outcome. It is known that explorative and coping strategies are relevant for depression, however these aspects are generally not considered in animal behavioral testing. Here we investigate the Flinders Sensitive Line (FSL), a rat model of depression, compared to the Sprague-Dawley (SD) rat in three independent tests where the animals are allowed to express a more extensive behavioral repertoire. The multivariate concentric square field™ (MCSF) and the novel cage tests evoke exploratory behaviors in a novel environment and the home cage change test evokes social behaviors in the re-establishment of a social hierarchy. In the MCSF test, FSL rats exhibited less exploratory drive and more risk-assessment behavior compared to SD rats. When re-exposed to the arena, FSL, but not SD rats, increased their exploratory behavior compared to the first trial and displayed risk-assessment behavior to the same extent as SD rats. Thus, the behavior of FSL rats was more similar to that of SDs when the rats were familiar with the arena. In the novel cage test FSL rats exhibited a reactive coping style, consistent with the reduced exploration observed in the MCSF. Reactive coping is associated with less aggressive behavior. Accordingly, FSL rats displayed less aggressive behavior in the home cage change test. Taken together, our data show that FSL rats express altered exploratory behavior and reactive coping style. Reduced interest is a core symptom of depression, and individuals with a reactive coping style are more vulnerable to the disease. Our results support the use of FSL rats as an animal model of depression and increase our understanding of the FSL rat beyond the behavioral dimensions targeted by the traditional depression-related tests.

## Introduction

Animal models are necessary to understand the genetic, environmental, and neurobiological underpinnings of neuropsychiatric disorders that studies in humans cannot sufficiently control for or access. The need for new experimental tests and improved animal models have been emphasized in order to gain progress in understanding pathophysiology of complex human disorders and to develop new treatment strategies (Nestler and Hyman, 2010; Anonymous, 2011). Depression is a broad diagnosis based on subjective symptoms of the patients. To date, we still do not understand the underlying mechanisms of depression, nor do we have reliable biomarkers. This has lead the National Institute of Health (NIH) to propose a framework to classify mental diseases based on functional constructs: the Research Domain Criteria (RDoC) (Cuthbert and Insel, 2013). Animal tests traditionally used in domains related to depression measure a single either-or behavior in response to a given, often stressful, stimulus. For example, in the forced swim test the time spent swimming vs. staying immobile is measured as the response to acute threat in the domain of negative valence, according to the RDoC. However, the exposure to stress evokes a spectrum of behavioral responses that is not taken into account in traditional tests (Kas et al., 2007), despite the knowledge that the response to novelty and coping styles in stress handling are important factors determining resilience or vulnerability to depression (Hankin, 2012). By expanding animal research beyond the use of traditional tests, this framework will facilitate the translation of experimental work into the clinic.

The Flinders Sensitive Line (FSL) is a rat model of depression. Over the years, behavioral studies on FSL rats have supported the face and predictive validity of the model in relation to human depression using traditional rodent tests for assessment of depressive-like behavior (Overstreet and Wegener, 2013). An alternative approach is to turn toward basal drives, such as exploration, risk assessment, risk taking, and shelter seeking, which are evolutionary conserved and therefore of importance from a translational perspective (Gosling, 2001; Sih et al., 2004). These aspects can be evaluated in a more complex setting where the animal is allowed to freely express a broad range of behaviors. In the multivariate concentric square field™ (MCSF) test, the animal is introduced to an arena including open areas, enriched zones to encourage exploration, sheltered areas, elevated passages, and different light conditions. This test was developed by Meyerson et al. and has been behaviorally validated with regard to identification of risky as opposed to safe areas (Meyerson et al., 2006). The MCSF test has already been used in several studies for behavioral profiling of selectively bred rat lines (Roman et al., 2007; Roman and Colombo, 2009). Another approach taking advantage of the animals' spontaneous behavior is to score continuous behaviors in a mild aversive environment such as a novel home cage without cage mates (Marques et al., 2008). The individual behaviors including exploring and passive/avoiding actions are grouped in categories based on emotional reactivity and stress coping styles (Steimer et al., 1997; Koolhaas et al., 1999; Marques et al., 2008). By including social challenges, the individual coping style can be assessed by observing the subtle dominance behavior, for example when animals re-establish their dominance-subordination relationships after a cage change in the home cage change test. The concept of stress coping styles was developed based on two opposite physiological responses to stress (Koolhaas et al., 1999). Proactive coping responses are characterized by territory control, high aggression, and a physiological response including increased blood pressure and adrenaline release. Conversely, reactive coping responses are characterized by immobility and low aggression, accompanied with decreased blood pressure and increased plasma levels of corticosterone (Koolhaas et al., 2007, 2011). Reactive coping style and submissive behavior are associated with depression-like behavior in animal models (Blanchard and Blanchard, 1990; Hasler et al., 2004; Nesher et al., 2013).

The aim of the present study is to achieve a more detailed and nuanced analysis of explorative strategies in a complex environment and of stress coping styles in FSL rats, which has not yet been performed. Here we show that when analyzed in the MCSF, the novel cage test and the home cage change test for social interaction, the FSL rats display an altered explorative strategy compared to SD rats and are characterized by reactive coping style.

## Materials and methods

### Animals and housing

Behavioral testing was performed on age-matched male Sprague-Dawley (SD) (Charles River Laboratories, Germany) and FSL rats (bred in-house, three different litters) between 12 and 14 weeks of age (*n* = 12/group, all SD rats came from different litters). SD rats (which the FSL was derived from) were used as control in order to compare the behavior of FSL rats to a well-characterized strain rather than another selectively bred line. The animals were housed under controlled room temperature (21 ± 2°C) and humidity (50%), with a reversed 12-h light/dark cycle (lights off from 12 p.m. to 12 a.m.). The rats were group-housed (two rats per cage) in transparent cages (1354G Eurostandard Type IV, Tecniplast, Italy) with bedding material (Aspen wood, Tapvei, Harjumaa, Estonia) and paper towels as enrichment. Food (R34 chow, Labfor, Lantmännen, Stockholm, Sweden) and water were provided *ad libitum*. All animal experiments were approved by the Stockholm North Committee on Ethics on Animal Experimentation and followed the guidelines of the Swedish Legislation on Animal Experimentation (Animal Welfare Act SFS1998:56) and the European Communities Council Directive (86/609/EEC).

### Procedure

**Figure 1:**
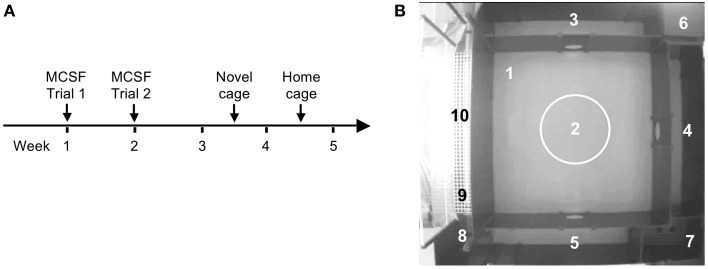
**The experimental outline (A) and the MCSF arena with the defined zones (B) numbered as follows: 1, center; 2, central circle (CTRCI); 3–5, corridors (corridors A–C); 6, dark corner room (DCR); 7, hurdle with the elevated hole board; 8, slope; 9, bridge entrance (BE); 10, bridge**.

The experimental outline is illustrated in Figure 1A. Animals were handled daily 5 days prior to the start of the first test. Handling consisted of individual handling, weighing, and adaptation to the transportation bucket that was used to carry the rat from the home cage to the testing room. All animals were weighed before the MCSF test and before the novel cage test. FSL rats had lower body weight than SD rats at both time points (Tables S1, S2). Reduced body weight of FSL rats is well-described (Overstreet, 1993).

The MCSF testing consisted of two trials 1 week apart. Seven to nine days after the second trial of the MCSF test, the rats were exposed to the novel cage test and a week later to the home cage change test. FSL and SD rats were alternated in order to avoid day-time and order bias: individual animals were tested in the same period of the day throughout the behavioral tests (±1 h) and the order of testing was maintained. All testing occurred during the dark period of the light/dark cycle, at least 1.5 h apart from the light switch, in a room where temperature, humidity, and background noise were similar to those in the housing room. The rats were video-recorded by a digital camera placed above the test apparatus and a person blinded to the experimental groups scored the behaviors.

### MCSF test

The MCSF test was performed as previously described in detail (Roman and Colombo, 2009). The arena consists of a large square field (100 × 100 cm) containing a smaller central square field (70 × 70 cm). The walls are 25 cm high except for the walls surrounding the bridge, which are 40 cm high. The entire arena is divided into zones (Figure 1B): center (#1): the square field in the middle of the arena; central circle (CTRCI, #2): the circular area (∅ = 22 cm) in the middle of the center; corridors A–C (#3–5): corridors surrounding the center; dark corner room (DCR, #6): the covered area providing shelter; hurdle (#7): an elevated passage with a hole board; the slope (#8): leading to the bridge; bridge entrance (BE, #9): the first part of the bridge before the light source; bridge (#10): the elevated and illuminated bridge construction. The approximate light conditions (in lux) were: center <25, CTRCI 30–35, corridors and hurdle <20, DCR <1 and bridge 600–850. Light intensity was measured before and after each daily session. The MCSF arena has been well-characterized to analyze exploratory behavior. Risk areas have been identified with the bridge and the central circle whereas the DCR has been shown to serve as a shelter: lactating rat dams retrieve their pups from the elevated and illuminated bridge to the DCR. Similarly, male rats collected food pellets from the bridge and hoarded and consumed the pellets in the DCR. Neither pups nor food pellets were moved out from the DCR when placed there (Meyerson et al., 2006). The areas immediately leading to the bridge (slope and bridge entrance) are used by the animal to assess the risk of visiting the bridge (risk assessment). Therefore, visits to those areas, together with the stretched attend postures, are considered as risk assessment.

The rat to be tested was transferred in a dark bucket from the home cage to the arena and released in the center facing the wall without openings. Each test session lasted 30 min. A short representative 5-min video is attached as Supplementary Material. The arena floor and walls were wiped with 10% ethanol solution between animals and sufficient time was allowed for the floor to dry before the next rat was placed in the arena.

The number of rearing actions, stretched attend postures (SAPs), grooming actions and nose pokes into the hole board of the hurdle were scored by direct observation. Urine spots and fecal boli were counted at the end of each trial. The EthoVision system (XT 10.0, Noldus Inc., Wageningen, Netherlands) was used, where the rat was detected by using the multiple body points setting with the tail base point required for counting. Latency (LAT, s) to first visit of each zone, number (referred to as frequency, FRQ) and duration (DUR, s) of the visits, distance moved (cm) and mean velocity (cm/s) within each zone were automatically detected. Measures for all the corridors (TOTAL CORR) and the total MCSF (ARENA) were derived. Calculated measures were: mean duration per visit to a zone (DUR/FRQ), relative frequency and duration as fraction of the total number and the total time of visits, number of rats visiting each zone or performing a particular behavior (occurrence, OCC). The descriptive parameters (Tables S1–S3) are grouped into functional categories on the basis of previous studies (Roman and Colombo, 2009; Meyerson et al., 2013). A list of abbreviations and indexes is shown in Table 1. Indexes are calculated from descriptive parameters for interpretation of impulsive-like behavior (slope/bridge interval) and anxiety-like behavior (risk/shelter indexes) as described in Table 1 (Roman et al., 2012). Here we have also introduced the shelter/corridor index as indicator of home base exploration (Table 1). Since the corridor A of the MCSF arena is the only access to the shelter, a rat that considers the shelter as a home base is more likely to enter into the DCR each time it enters the corridor A, providing us with a measurement for home base exploration. Finally, descriptive parameters within the same or similar functional category are taken into account in the trend analysis used to reveal an overall behavioral profile (Meyerson et al., 2013).

**Table 1:** **Abbreviations, indexes and trend analysis categories used in the interpretation of behaviors from the multivariate concentric square field ™**(MCSF)** test**.

**PARAMETERS**	
FRQ = frequency (number of visits)	
%FRQ = frequency as percent of the total number of visits to all zones	
DUR = duration (s)	
DUR/FRQ = time (s) per visit	
LAT = latency (s)	
OCC = occurrence (the number of rats visiting each zone or performing a particular behavior	
SAP = stretched attend posture	
TOTAL ACT = number of visits to all zones	
**ZONES**	
CENTER = central field	
CTRCI = central circle	
CORR = corridor	
DCR = dark corner room (shelter)	
BE = bridge entrance	
**INDEXES**	
*Shelter/corridor index* = (FRQ DCR − FRQ corridor A)/(FRQ DCR + FRQ corridor A), measuring the recourse to the DCR on the total visits to the DCR and the corridor A. It indicates how much the shelter was considered as home base for exploration. Thus, a value close to zero indicates higher DCR base exploration.	
*Slope/bridge interval* = (LAT slope − LAT bridge)/(LAT slope), indicating the delay of visiting the bridge from the slope; used as one way of interpreting impulsive-like behavior. Thus, a value close to zero indicates less risk-assessment and faster risk-taking response.	
*Risk/shelter duration index* = (DUR bridge − DUR DCR)/(DUR bridge + DUR DCR). *Risk/shelter frequency index* = (FRQ bridge − FRQ DCR)/(FRQ bridge + FRQ DCR), measuring the performance on the bridge compared to the DCR; used as one way of interpreting anxiety-like behavior. Thus, a higher value indicates that the animal accessed the bridge more than the DCR and is interpreted as lower anxiety-like behavior.	
**TREND ANALYSIS**	
**Functional category**	**Parameters**
General activity	Total act, FRQ and (inv)DUR/FRQ total corridors, FRQ center, distance arena
Exploratory activity	(inv)DUR total corridors, (inv)DUR center, DUR hurdle, rearings, nose pokes
Shelter seeking	FRQ, DUR, and DUR/FRQ DCR
Risk assessment	Stretched attend postures, DUR/FRQ slope and bridge entrance
Risk taking	FRQ, DUR, and DUR/FRQ bridge; FRQ, DUR, and DUR/FRQ central circle

*The parameters indicated by (inv) were inversely ranked since they are negatively related to the functional category*.

### Novel cage test

The novel cage test was originally developed for mice to assess individual exploratory- and risk assessment-related behaviors (Marques et al., 2008). For the present study the test was adapted for rats to have a symmetric arena (40 × 40 × 40 cm, made of dark gray plastic) where the walls allowed the rat to perform rearings and protected from external visual cues (Steimer et al., 1997). The exploratory behaviors exhibited in the test were interpreted as indicators of emotional reactivity and coping style (Steimer et al., 1997; Koolhaas et al., 1999; Marques et al., 2008). The rat was released in the center of the arena, facing the left wall, under dimmed light (approximately 25 lux; similar to the light conditions used in the center of the MCSF arena). Each test session lasted 5 min. The cage was cleaned with 10% ethanol solution between animals. Frequency (total number of each behavior per session) and duration (total time of each behavior per session, in seconds) of the individual behaviors described in Table 2 were manually scored with EthoLog® (Ottoni, 2000), and relative frequency and duration were calculated as fractions of the total behavior scored per rat. Individual behaviors were grouped into functional categories based on stress coping style previously described (Fernandez-Espejo and Mir, 1990; Steimer et al., 1997; Koolhaas et al., 1999) (Table 2). The scores for coping styles were calculated as the sum of relative frequency or duration of the individual behaviors grouped within the same functional category.

**Table 2:** **Ethograms of behaviors scored in the novel cage and the home cage change tests**.

**NOVEL CAGE TEST**		
**Functional category**	**Individual behaviors**	**Description**
Proactive coping	Stretched approach	Walking with a flat body posture stretched and close to the floor
	Stretched attend posture	The rat stretches the neck or front part of the body forwards with four paws on the floor and then returns backwards
	Grooming	Scratching, shaking, wiping, or licking body parts (fur, ears, nose, and tail)
Reactive coping	Freezing	Sudden suppression of movements
	Motionless	Sitting or lying without suppression of movements
Exploratory activity	Free rearing	Standing on hind legs
	Investigating	Exploring the floor, cage walls, or air through olfactory activity
Locomotor activity	Wall rearing	Standing on hind legs with forepaws leaning against a wall
	Walking	Locomotor behavior with normal body posture
**HOME CAGE CHANGE TEST**		
**Functional category**	**Social behaviors**	**Description**
Neutral behavior	Head–head	The head of the rat touches the head of the other rat
	Nose–side	The rat sniffs between the ventral region and the back of the other rat
	Nose–nose	The rat sniffs the other rat's nose in an equal sniff
	Passing	The rat passes the other rat either in a direct meeting or from behind
Dominant behavior	Head–tail	The head of the rat touches the tail of the other rat
	Nose–genitals	The nose of the rat touches the genitals of the other rat
	Following	The rat follows the other rat for more than two steps
	Approaching	The rat is walking or running more than three steps without hesitation with the nose pointed in direction toward the other rat and fulfills the approach with a minimum of 5 cm away from the other rat
	Nuzzling	The rat sniffs/bites/grooms the other rat in the area between the tip of the nose and the ventral region
	Mount 1	The rat rears and leans its front legs on the other rat's back from behind
Aggressive behavior	Mount 2	The rat rears and leans its front legs on the other rat's back from behind and makes copulation movements
	Chasing	The rat runs after the other rat for more than two steps
	Fight	Very rapid rolling, jumping, and biting of both animals while being in close contact
Submissive behavior	Avoiding	The rat moves or faces in a direction away from the other rat when the other rat is approaching
	Crowing under	The rat is crawling under the other rat
	Submissive posture	The rat is lying on its back with the other rat standing and/or leaning over its ventral part

### Home cage change test

The home cage change test was performed to examine subtle dominance-subordination relationships in a home cage. Group-housed rats were transferred into a new home cage (1354G Eurostar Type IV, Techniplast, Italy) with bedding material (Aspen wood, Tapvei, Harjumaa, Estonia) in order to simulate a cage change, and were video-recorded for 10 min under red light of about 3 lux. The rats were marked with a pen on their back for identification. Shuffling of bedding in front of and beside them was scored as burrowing behavior and was considered as defensive behavior (Dudek et al., 1983). Frequency (total number of each behavior per session, in bouts) and duration (total time of each behavior per session, in seconds) of the social behaviors described in Table 2 were manually scored with EthoLog® (Ottoni, 2000), and relative frequency and duration were calculated as fraction of the total social behavior scored per rat. Social behaviors were grouped into functional categories as previously described (Koolhaas et al., 1980; Fernandez-Espejo and Mir, 1990) (Table 2). The scores for the social behavior categories were calculated as the sum of relative frequency or duration of the social behaviors grouped within the same functional category.

### Statistical analyses

The majority of the behavioral data were not normally distributed according to the D'Agostino and Pearson omnibus normality test. Therefore, the statistical analysis was run by non-parametric tests. The Mann-Whitney U-test was used for comparison between experimental groups and the Wilcoxon matched pairs test was used for within-group comparison between the first and the second MCSF trial. When the rat did not enter a particular zone of the MCSF, values of dependent variables relative to that zone were considered missing, except for comparison between trials where duration and frequency measures were given as zero and latency measures were set to 1800 s (session duration). Intra-trial time courses were analyzed with the Friedman One-Way ANOVA followed by Dunn's *post-hoc* test. For the indexes, an absent value was considered as missing data. The Fisher's exact test was used for analysis of occurrence. The Spearman's rank correlation test was used to check for monotonic relationships between paired data.

For analysis of behavioral profiles in the MCSF test, a trend analysis ranking procedure was used as previously described (Meyerson et al., 2013). Briefly, rats are ranked across experimental groups and trials for each parameter, and the ranking values for parameters within each functional group are summed (Table 1). Statistical analysis tested the hypothesis that experimental group or trial influences rat behavior and therefore its rank position. Since the results from the ranking were normally distributed, comparison of within-trial trend analysis was done by unpaired *t*-test, and repeated trial and interaction effect was tested by Two-Way repeated measures ANOVA with Bonferroni *post-hoc* analysis.

Differences were considered statistically significant at *p* ≤ 0.05 and statistical trends (*T*) were defined as 0.05 < *p* ≤ 0.1. The JMP 11 (SAS Institute Inc., NC, USA) and Prism 5 (GraphPad software Inc.) were used for statistical analysis.

Multivariate data analysis procedures, i.e., the Soft Independent Modeling of Class Analogy (SIMCA), were used to complement conventional statistical analyses. The principal component analysis (PCA) was use to extract and display the systemic variation in the novel cage test data from the MCSF test. The relative frequency and duration of behaviors were included in the analysis (Table S4). In the PCA, variables were pre-processed by scaling and mean-centering in order to standardize weighting of each parameter. The first component in the PCA represents the largest variation in the data set, the second component the largest of the remaining variance, etc. The PCA creates a score plot showing a summary of the relationships among the individuals, e.g., how individuals cluster in groups, and a loading plot identifying variables important for creating these relationships, i.e., behavioral parameters. The direction of the score plot corresponds to the direction in the loading plot (Jackson, 1991) (Eriksson et al., 2006).

PLS-DA is a regression extension of PCA and calculates the relationship between a Y-matrix (here experimental groups, i.e., SD and FSL rats) and an X-matrix (here MCSF parameters). The weights for the X-variables (in the analysis denoted w) indicate the importance of these variables, while the weights for the Y-variables (in the analysis denoted c) indicate which Y-variables are modeled in the respective PLS model dimensions. When these coefficients are plotted, a picture showing the relationships between X and Y is obtained (Eriksson et al., 2006). The two groups will locate opposite to each other and the parameters most characteristic for the respective group will load close to that group. The PLS-DA was used for analysis of performance in the first MCSF trial. The SIMCA 13.0 software (Umetrics®, Umeå, Sweden) was used.

## Results

### The first MCSF test (novelty)

**Figure 2:**
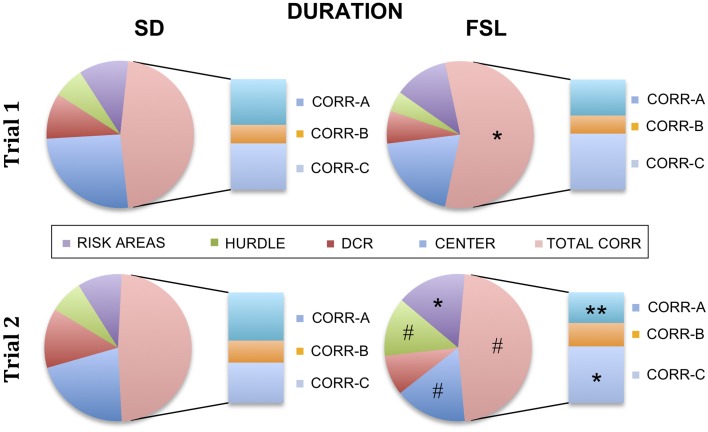
**Percent of time spent in each zone in the MCSF in relation to the total trial time for SD (left) and FSL rats (right), in trial 1 (top) and in trial 2 (bottom)**. The risk areas include slope, bridge entrance, bridge, and central circle. The stacked bar inserts show the time spent in the corridors A–C as percent of the total time spent in all corridors. ^*^p < 0.05, ^**^p < 0.01 Mann-Whitney U-test for comparison of FSL vs. SD rats; ^#^*p* < 0.05 Wilcoxon matched pairs test for within-group comparison of trial 2 vs. trial 1.

**Figure 3:**
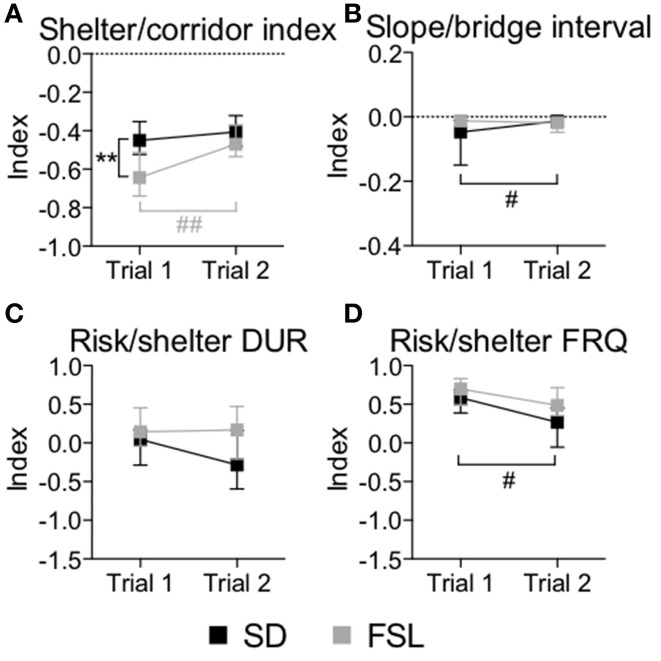
**Indexes used for interpretation of performance in trial 1 and trial 2 in the MCSF test in SD and FSL rats**. Values are shown as median and interquartile range for the following indexes: shelter/corridor index used for home base behavior **(A)**, slope/bridge interval for interpretation of impulsive-like behavior **(B)**, and risk/shelter duration **(C)** and frequency **(D)** indexes used for interpretation of anxiety-like behavior. ^**^p < 0.01 Mann-Whitney U-test for comparison of FSL vs. SD rats; ^#^*p* < 0.05, ^##^*p* < 0.01 Wilcoxon matched pairs test for within-group comparison of trial 2 vs. trial 1 (the black symbol # refers to the SD group, the gray symbols ## to the FSL group).

The descriptive parameters from the first trial of the MCSF are displayed in Table S1 and Figure 2. In the category general activity, FSL rats covered shorter distance in the center compared to SD rats. Furthermore, FSL rats were slower than SD rats in the corridors (Table S1). In exploratory activity, FSL rats spent longer time in the corridors (Figure 2, Table S1) and less time per visit in the hurdle (Table S1) compared to SD rats. Shelter-seeking associated parameters differed between the groups, where FSL rats made fewer visits to the DCR than SD rats (Table S1). Moreover, risk-assessment behavior differed between groups, with FSL rats displaying more stretched attend postures than SD rats (Table S1). For the descriptive measures with relevance for risk-taking behavior, FSL rats differed from SD rats by a shorter latency to first visit of the central circle and a decreased velocity in the central circle (Table S1). In agreement with fewer visits to the DCR, the shelter/corridor index in FSL rats was lower than in SD rats (Figure 3A). The indexes of impulsive-like and anxiety-related behaviors (slope/bridge interval and risk/shelter indexes, respectively) did not differ between FSL and SD rats (Figures 3B–D).

The pattern of behavior is further illustrated in the PLS-DA analysis (Figure S1). The parameters that were significantly different between the groups loaded close to the group where they contributed most to the model. In addition, parameters with a statistical trend in the traditional statistical analysis (marked with a “T” in Table S1) loaded close to the SD rats. Moreover, FSL rats were characterized by generally longer, although not statistically significant, latency measures relative to SD rats (Table S1) and in the PLS-DA all latencies except the latency to first visit the central circle are located on the same side as the FSL rats.

**Figure 4:**
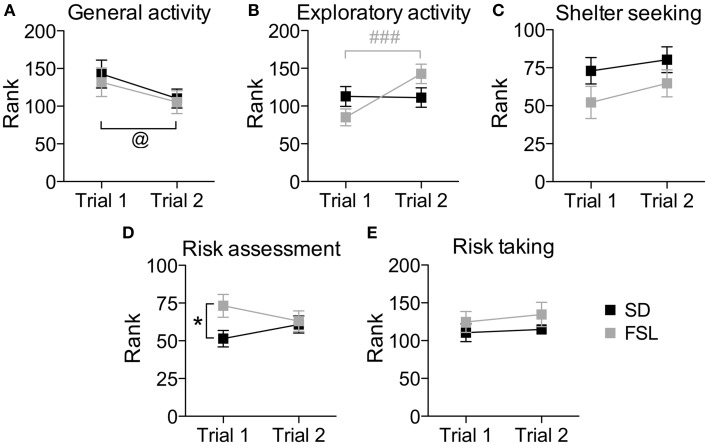
**Trend analysis of behavioral profiles in trial 1 and trial 2 in the MCSF test in SD and FSL rats**. Values are shown as mean ± SEM for the functional categories general activity **(A)**, exploratory activity **(B)**, shelter seeking **(C)**, risk assessment **(D)**, and risk taking **(E)**. Repeated measures ANOVA was used to test for repeated trial and trial x group interaction. ^@^*p* < 0.05 main effect of repeated trial, ^###^*p* < 0.001 Bonferroni *post-hoc* test for within-FSL group comparison of trial 2 vs. trial 1 (trial × group interaction and repeated trial effect). ^*^p < 0.05 unpaired *t*-test for within-trial comparison of FSL vs. SD rats.

The overall behavioral profile shown by the trend analysis (Figure 4), in which the individual strategies within the same context are taken into account, revealed higher risk assessment and a trend (*p* = 0.078) for lower shelter-seeking behavior in FSL compared to SD rats.

**Figure 5:**
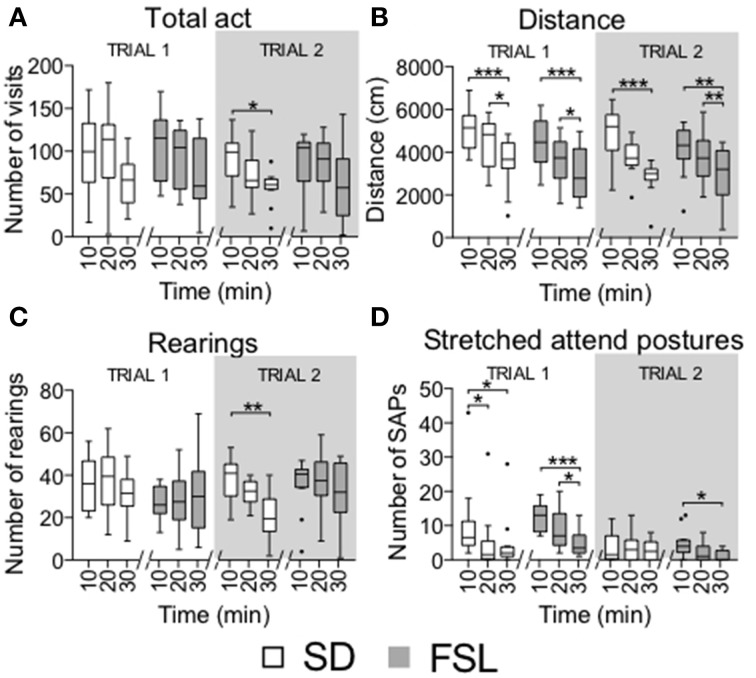
**Time course of activity measures by 10-min bins in trial 1 and trial 2 in the MCSF test in SD and FSL rats**. Values are represented by box (median and interquartile range) and Tukey whiskers plots for number of visits to all the arena zones **(A)**, distance covered in the total arena **(B)**, number of rearings **(C)**, and stretched attend postures **(D)**. ^*^p < 0.05, ^**^p < 0.01, ^***^p < 0.001 Dunn's *post-hoc* test for within-group and within-trial comparison of 10-min bins.

Habituation to the arena, shown as the total number of visits (total act) and distance in the total arena, rearings and stretched attend postures per 10-min bins (Figure 5), was similar in FSL and SD rats. Both groups had a reduction in distance covered and stretched attend postures during the 30-min trial [distance FSL: *F*_(2, 22)_ = 18.5, *p* < 0.0001; SD: *F*_(2, 22)_ = 14, *p* = 0.0009; SAPs FSL: *F*_(2, 22)_ = 17.9, *p* < 0.0001; SD: *F*_(2, 22)_ = 10.8, *p* = 0.0044].

### The repeated MCSF test (trial 2)

The descriptive parameters from the second trial in the MCSF are displayed in Table S2 and Figure 2. In the general activity category, FSL rats covered shorter distance in the center than SD rats (Table S2). No differences between FSL rats and SD rats were found among descriptive parameters associated with exploratory activity (Table S2). For shelter seeking-related parameters, FSL rats displayed longer latency to reach the DCR and made fewer visits than SD rats (Table S2). Accordingly, the percent of time spent in the corridor A, i.e., the access way to the DCR, was lower in FSL compared to SD rats (Figure 2). No difference was found in the shelter/corridor index (Figure 3A). In measures associated with risk assessment and risk taking, FSL rats spent longer time than SD rats in the bridge entrance (Table S2) and in the risk areas in proportion to other zones (Figure 2). Accordingly, the percent of time spent in corridor C was higher in FSL rats compared to SD rats, since corridor C is the access way to the bridge (Figure 2). Both the slope/bridge interval and the risk/shelter indexes did not significantly differ between groups (Figures 3B–D).

The overall behavioral profile shown by the trend analysis (Figure 4) revealed no significant differences between the groups.

Time course in 10-min bins in trial 2 showed that FSL rats displayed habituation in the distance covered to the same extent as SD rats. The number of stretched attend postures decreased over time only in FSL rats. In SD rats, the number of visits, distance and rearings decreased over time [total act SD: *F*_(2, 22)_ = 6.5, *p* = 0.0388; distance FSL, *F*_(2, 22)_ = 12.5, *p* = 0.0019; SD, *F*_(2, 22)_ = 13.5, *p* = 0.0012; rearings SD, *F*_(2, 22)_ = 11.4, *p* = 0.0034; SAPs FSL, *F*_(2, 22)_ = 9.8, *p* = 0.0074; Figure 5].

### Differences in the familiarization to the MCSF (trial 2 vs. trial 1)

The difference between a familiar (trial 2) and a novel (trial 1) environment with regard to descriptive parameters is displayed in Table S3 and Figure 2. Many of these differences are to be expected and prove that the animals recognize the arena and have established a memory from the first trial. Indeed, general activity was lower in both groups in the second compared to the first trial [trial effect *F*_(1, 22)_ = 8.5, *p* = 0.008, Figure 4A]. SD rats showed a decrease in the risk/shelter index (interpreted as more anxiety-related behavior) and an increase in the slope/bridge interval index (interpreted as higher impulsive-like behavior) in the second vs. the first trial, while no significant difference was found for FSL rats (Figures 3B–D). The index related to the home base behavior (shelter/corridor index) increased in FSL but not in SD rats in trial 2 compared to trial 1 (Figure 3A). Despite this increase in the use of the shelter, the trend analysis showed that FSL rats tended (*p* = 0.084) to display less shelter-seeking behavior than SD rats. The main finding was an overall increase in exploratory activity in FSL rats in the second compared to the first trial, in contrast to no change in SD rats. Indeed, the trend analysis showed a strain × trial interaction [*F*_(1, 22)_ = 15.7, *p* = 0.0007] and trial effect [*F*_(1, 22)_ = 14.0, *p* = 0.0011] (Figure 4B).

### Novel cage test

**Figure 6:**
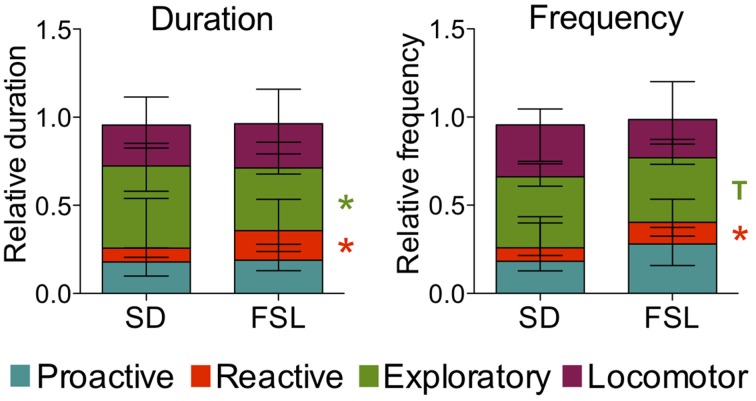
**Coping styles assessed using the novel cage test in SD and FSL rats**. Duration **(left)** and frequency **(right)** of the coping styles are expressed as fraction of the total behavior scored per rat. Data are presented as median and interquartile range. T, trend (0.05 < *p* ≤ 0.1); ^*^p < 0.05 Mann-Whitney U-test for comparison of FSL vs. SD rats.

The descriptive parameters from the novel cage test are displayed in Table S4. In the behaviors associated with reactive coping, both duration and frequency of motionless were higher in FSL than in SD rats. In measures related to exploratory activity, both duration and frequency of investigating behavior were lower in FSL than in SD rats (Table S4). In agreement, the comparison of the scores for coping styles revealed that frequency and duration of the reactive coping style were higher in FSL rats compared to SD rats (Figure 6). The exploratory coping duration was significantly shorter in FSL compared to SD rats. No differences between groups were found for the proactive and the locomotor coping styles (Figure 6).

The reactive coping style data were partly confirmed by the PCA (Figure S2). In the score plot, FSL rats were mostly located in the upper right and center, whereas SD rats had a more spread-out location in the lower right and upper left quadrants, but the two groups were not completely separated. The FSL group localization in the score plot (Figure S2A) corresponded to higher frequency and duration of motionless, characteristic of a reactive coping style, and stretched attend postures (Figure S2B). The investigative behavior, that was lower in the FSL rats, loaded opposite to motionless. The two principal components explained 51% of the variance (*R*^2^*X* = 0.51; *Q*^2^*X* = −0.02) and values of explained variation and predicted variation were within a range previously observed for biological data (Roman and Colombo, 2009; Lundstedt-Enkel et al., 2010; Meyerson et al., 2013; Palm et al., 2014).

### Home cage change test

**Figure 7:**
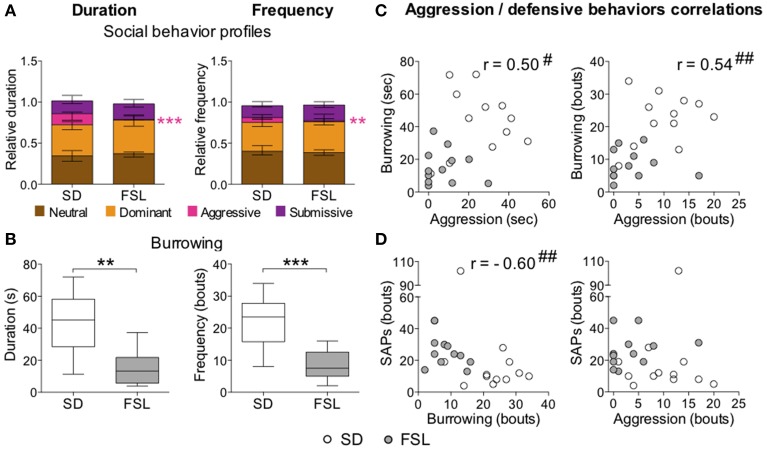
**Social behaviors (A) and burrowing (B) in the home cage change test in SD and FSL rats, and correlations between aggression and the defensive behaviors burrowing and SAPs (C,D)**. Duration (left) and frequency (right) of the social behavior categories **(A)** are expressed as fraction of the total behavior scored per rat. Data are presented as median and interquartile range. Burrowing duration (s) and frequency (bouts) **(B)** are represented by box (median and interquartile range) and Tukey whiskers plots. Aggression, burrowing, and SAPs performed by SD and FSL rats are plotted and tested for correlations **(C,D)**; aggression vs. burrowing duration **(C**-left**)** and frequency **(C**-right**)**, burrowing vs. SAP frequency **(D**-left**)**, aggression vs. SAP frequency **(D**-right**)**. SAPs measures derive from the first trial of the MCSF test. ^**^p < 0.01, ^***^p < 0.001 Mann-Whitney U-test for comparison of FSL vs. SD rats; ^#^*p* < 0.05, ^##^*p* < 0.01 Spearman rank correlation tests, with the correlation coefficient *r*.

In the home cage change test, there was no difference between the groups in the total score for duration and frequency of all social behaviors (data not shown). The descriptive parameters from the home cage change test are displayed in Table S5 as relative duration and frequency values. No major differences were observed in the category of neutral behaviors. In the category of dominant behaviors, both duration and frequency of the parameter mount 1 were higher in FSL than in SD rats, while the nuzzling measures were lower in FSL than in SD rats. With regard to aggressive behaviors, FSL rats exhibited less fighting than SD rats (in duration, frequency, and occurrence). Notably, in the submissive behavior category, FSL rats exhibited less submissive postures (duration, frequency, and occurrence) and more avoiding behaviors (both in duration and frequency) compared to SD rats. In the overall analysis of social behaviors, FSL rats displayed less aggressive behavior (Figure 7A) compared to SD rats, but no net differences in the category of neutral, dominant, and submissive behaviors. Besides less aggression, FSL rats also exhibited less defensive behavior burrowing (Figure 7B) compared to SD rats. Conversely, stretched attend postures are a risk-assessment behavior related to emotionality. Group difference in stretched attend postures was observed in the novel MCSF, where this behavior is possibly evoked to a larger extent than in a more simple context as the novel cage (see Discussion) (Grewal et al., 1997). We wanted to assess the relations between stretched attend postures performed in the MCSF, the defensive-related behavior burrowing and the social aggression exhibited in the home cage change test. Testing for monotonic relationship revealed the following correlations: aggression–burrowing (both duration and frequency), moderate positive correlation (0.40 ≤ |*r*| ≤ 0.59), with FSL rats clustered with low aggression and low burrowing and SD rats clustered at the opposite values (Figure 7C); burrowing–stretched attend postures, strong negative correlation (0.60 ≤ |*r*| ≤ 0.79), with FSL rats clustered with low burrowing and high stretched attend postures and SD rats clustered around the opposite values (Figure 7D-left); aggression–stretched attend postures: no correlation (Figure 7D-right). Thus, characteristic features of FSL rats were low aggression, burrowing, and high frequency of stretched attend postures.

## Discussion

FSL rats were tested in a multivariate environment, i.e., the MCSF arena, in order to evoke a broad behavior repertoire. The descriptive parameters together with the PLS-DA indicated slightly altered explorative strategies, and the trend analysis revealed higher risk-assessment behavior in FSL relative to SD rats in the first trial. When the rats were re-exposed to the arena in a second trial, both groups displayed reduced general activity, as expected in a familiar environment. Surprisingly, FSL rats, but not SD, increased their exploratory activity and changed their home base strategy. Notably, FSL risk-assessment behavior in trial 2 did not differ from SD rats. Taken together, the behavior of FSL rats was more similar to that of SD rats when re-exposed to the arena compared to the first trial. Moreover, coping styles related to exploration of a novel environment were assessed by the novel cage test and social behaviors were evaluated using the home cage change test. In good agreement with the MCSF results, FSL rats displayed more reactive coping and less exploratory behavior compared to SD rats when assessed in the novel cage test. In support of a reactive coping style, FSL rats were also characterized by less aggressive behavior in comparison with SD rats in the home cage change test.

### FSL explorative strategy in a novel environment

Low novelty-induced and goal-directed exploration has been observed in FSL rats in previous studies using the open field and the novel object recognition tests, respectively (Overstreet, 1986; Gomez-Galan et al., 2013). In the novel MCSF arena, FSL rats were active and explored all zones of the arena in a fashion similar to that of SD rats. However, subtle differences between FSL and SD rats were found (Table S1). FSL rats were characterized by more time spent in the corridors and lower velocity. These results together with generally longer latency measures in FSL rats indicate a slightly lower exploratory drive compared to SD rats, in line with the previous finding that FSL rats display hypoactivity (Kokras et al., 2011). In the MCSF, the rat could choose between three corridors to leave the center and the choice of corridor affects the time for reaching the other zones. Therefore, a lack of a statistical difference in latency to the first visit of the corridors was to be expected and proved that there was no bias between the groups in choosing corridor. Exploration of a novel environment derives from the balance between risk/benefit evaluation and novelty-seeking drive (Hughes, 1997) that may regulate the use of shelter- and risk-associated areas. In the MCSF, it has been shown that the bridge is associated with risk, the DCR with shelter (Meyerson et al., 2006), and lower exploration with an increased use of the DCR (Meyerson et al., 2006; Roman et al., 2007). Surprisingly, FSL rats tended to recur less to the shelter and preferred to stay more in the corridors compared to SD rats. This was not accounted for by their reduced exploration of the arena, since the percent of visits to the DCR was still lower in FSL than in SD rats (Table S1). Rodent explorative strategies in a novel environment include the identification of a home base, i.e., a secure place where rodents often recur after each excursion into the novel territory (Eilam and Golani, 1989). The corridor A of the MCSF arena is the only access to the shelter. Therefore, the rat that considers the shelter as home base is more likely to enter into the DCR each time it enters the corridor A. The shelter/corridor index was lower in FSL compared to SD rats in the first MCSF trial (Figure 3A), suggesting that FSL rats adopted a different strategy and did not use the shelter as a home base, as SD rats did. A similar strategy has previously been described in selectively bred Sardinian alcohol-preferring rats (Roman and Colombo, 2009), however accompanied by much lower general activity, exploratory drive and risk-taking behavior than that observed in FSL rats.

### Altered risk-assessment strategy in FSL rats

Risk assessment is considered a behavioral strategy to evaluate the potential risks vs. benefits deriving from exploration (Blanchard and Blanchard, 1988). A common measure of risk assessment is the stretched attend posture. This behavior has been interpreted as an indicator of anxiety-like behavior since it was affected by anxiolytic and anxiogenic drugs (Bickerdike et al., 1994; Shepherd et al., 1994). FSL rats displayed more stretched attend postures and higher general risk-assessment behavior than SD rats in the first MCSF trial (Figure 4D). In addition to the bridge, the most central part of the open center, i.e., the central circle, is considered a risk area and therefore both zones are associated with risk-taking behavior (Meyerson et al., 2006, 2013). FSL rats had a shorter latency to the first visit of the central circle, where they also moved slower than SD rats. This indicates less avoidance of the open area, in contrast to the increase in risk-assessment behavior. However, in general FSL rats performed similar to SD rats in the risk areas. These observations are in agreement with previous studies where the expression of stretched attend postures did not correlate with risk-taking behavior measured as time spent in the open arm of the zero-maze test (Bickerdike et al., 1994; Shepherd et al., 1994). The fact that FSL rats did not differ from SD rats in risk-taking behavior is in agreement with the result from the risk/shelter index. This index is used for interpretation of anxiety-like behavior as it captures the performance in the risk area bridge in relation to the sheltered DCR. No difference between FSL and SD rats was found in this index (Figures 3C,D). In addition, the lower recurrence to the DCR and the efficient performance in the risk areas, supported by the overall behavioral profiling revealed by the trend analysis, indicate that FSL rats are not characterized by elevated anxiety-like behavior compared to SD rats, but by increased emotionality displayed with stretched attend postures in the MCSF and with reactive coping style in the novel cage test.

### Reactive coping style of FSL rats is associated with low aggression

In the novel cage the challenge to explore is lower than in the MCSF, due to the absence of environmental enrichment. We suggest that this condition makes the expression of the animal behaviors more strongly driven by the animal internal stimulus or status compared to an enriched environment, and therefore it is suitable to investigate coping styles. In the novel cage test FSL rats displayed high immobility, either actively as in freezing or passively as in motionless, in agreement with unpublished separate observations in our lab. These behaviors can be summarized as reactive coping style and were higher in FSL rats compared to SD rats (Figure 6).

Reactive coping is also associated with low levels of aggression (Koolhaas et al., 1999). Social dominance behaviors were investigated in the home cage change test (Table S5). FSL rats exhibited less fighting and accordingly less submissive postures compared to SD rats, instead they displayed avoidance and a light version of mounting (indicated as mount 1 in Table 2). The reduced aggressive behavior displayed by the FSL rats in the home cage change test is in agreement with the reactive coping style assessed in the novel cage test. Burrowing has been described as an expression of defensive behaviors, shelter building for nesting, refuge and food storage (Bouchard and Lynch, 1989; Deacon, 2006) and can be considered as a displacement behavior for aggression when aggression is not suitable or possible to display, e.g., for a subordinate toward a dominant individual. Indeed, aggression and burrowing were positively correlated (Figure 7C), and a low expression of those behaviors characterized FSL rats. Stretched attend postures have been described as a risk-assessment strategy acted to search for and acknowledge potential threats (Blanchard and Blanchard, 1989), and related to high emotionality. In addition, when the threat is anticipated or recognized, either proactive defensive behaviors (i.e., burrowing and fighting) or reactive strategies (i.e., flight or hiding) can take place. The stretched attend postures counted during the first MCSF trial negatively correlated with the burrowing performed in the home cage change test (Figure 7D). Taken together, these data suggest that FSL rats display high emotionality in the potential presence of threats and the high emotionality is preferentially expressed through reactive rather than proactive responses (burrowing).

### Repeated testing and coping style in FSL rats

Using repeated exposure to the MCSF arena it has previously been shown that rats remember and can recall the arena (Meyerson et al., 2006; Roman et al., 2007; Roman and Colombo, 2009). Therefore, FSL rats were tested twice in the MCSF in order to study their ability to adapt and change their behavioral strategy when re-exposed to the same context. In the second trial, FSL rats, but not SD, altered their explorative activity (Figure 4B) and home base strategy (Figure 3A) compared to the first trial. Moreover, the group differences in stretched attend postures and risk assessment that were found in the first (novel) MCSF trial were absent during the second trial (Figure 4D). These findings suggest that FSL and SD rats make a different use of the previous experience of the arena. Notably, in addition to increased exploratory activity, FSL rats spent longer time in risk areas relative to other zones compared to SD rats in the second compared to the first trial (Figure 2). This further supports a lack of anxiety-related behaviors in a multivariate environment. It has previously been demonstrated that the initial behaviors of selectively bred Alko non-alcohol rats, interpreted as anxiety-related behaviors, became even more pronounced in a second, repeated MCSF trial exemplified by lower risk-taking behavior, shorter latency to first visit of the DCR and longer time spent in the DCR in the second trial (Roman et al., 2007). In contrast, the FSL rats do not display anxiety-like behavior in either the first or the second trial, underscoring the absence of anxiety-related behavior. Moreover, the increase of the exploration in FSL rats in the second MCSF trial further supports a reactive coping style. Indeed, low aggression levels and a reactive coping style have been associated with flexible explorative strategies and maintained exploratory behavior in response to small context changes (Benus et al., 1991). One interpretation is that the reactive coping style of FSL rats accounts for the larger difference in MCSF performance between trials 1 and 2 compared to SD rats. This larger difference had as a consequence that in the familiar MCSF the behavioral profile of FSL rats was similar to that of SD rats.

### Risk-assessment strategies and coping styles influence the interpretation of anxiety-related tests

Anxiety is often, but not always, associated with depression (Hirschfeld, 2001). We have previously observed that FSL rats exhibit increased anxiety-like behaviors in a number of traditional paradigms (Femenia et al., 2015). Instead, other groups have reported that FSL rats display less (Abildgaard et al., 2011) or no (Schiller et al., 1991) anxiety-related behavior (Overstreet, 1986; Schiller et al., 1991; Overstreet et al., 2004; Abildgaard et al., 2011). Our present study suggests that FSL rats do not display increased anxiety-like behavior in the multivariate arena. It is important to consider that behavioral patterns displayed in a multivariate setting differ from those exhibited in a setting with limited number of choices (an either-or situation). For example alcohol Preferring rats have shown characteristics of increased anxiety-like behavior compared to alcohol non-preferring rats in traditional tests (Stewart et al., 1993), while the performance in the MCSF test was characterized by higher risk-taking behavior of Preferring compared to non-preferring rats (Roman et al., 2012). For the FSL rats, differences in risk-assessment strategies may be taken into account. Risk-assessment behavior is more relevantly evoked by a complex novel environment, like the MCSF arena or the canopy stretched attend posture apparatus (Grewal et al., 1997), than the open field or the novel cage test. It is possible that if the test gives the rat the opportunity to explore areas of different qualities and encourages risk assessment, such as the MCSF area (Meyerson et al., 2006, 2013), the increased risk assessment of the FSL rats allows them to perform risk-taking behavior to the same extent as the SD rats. Also the expression of coping styles may be affected. Indeed, the reactive coping style of the FSL rats (characterized by motionless and reduced investigation in the novel cage) emerges in the MCSF arena as reduced exploratory drive in the exploration of a novel environment and flexibility when familiarized to the arena, as described above, with an increase of exploratory activity in the second compared to the first trial.

### The FSL rat as a model for depression

Depressed patients have a reduced explorative drive for novelty, and novelty seeking inversely correlates with the severity of depressive symptoms (Hansenne et al., 1999). Likewise, proactive coping is associated with decreased risk for depression, whereas avoidance and reactive coping enhance the risk (Nagase et al., 2009; Cairns et al., 2014; Roohafza et al., 2014). However, these aspects of depression are rarely described in animal models of depression. Here we try to translate these symptoms by assessing explorative strategies in a novel complex environment, adaptation when the environment is familiar, and coping styles in the FSL model of depression. Using three independent tests based on explorative and social strategies we show that FSL rats have a reduced explorative activity that could be due to an altered strategy of exploration. This is in agreement with findings showing that rats selected for persistently low exploratory activity display increased immobility time in the forced swim, but also anxiety-related behaviors in the elevated plus maze test (Mallo et al., 2007).

The profiling of the animals revealed that FSL rats were characterized by reactive coping style, including social dominance behaviors associated with low levels of aggression. Coping styles affect the behavioral responses to stress, testing situations, and pharmacological treatments. Indeed, selectively bred low-aggressive mice displayed reactive responses to the open field (similarly to what we observed in the FSL rats during the novel cage), increased immobility in the forced swim test and a different responsiveness to stressors and serotonergic drugs compared to high aggressive mice (Veenema et al., 2003, 2005). Our ethological observations illustrate coping-related behavioral features of FSL rats that complement our understanding of their behavior displayed in traditional depression-related tests and may be important to understand factors of susceptibility/resilience to stress.

## Conclusion

Using a multivariate behavioral approach we demonstrated that the exploratory behavior of FSL rats is not markedly different from that of SD rats. However, subtle differences exist. In a novel environment, FSL rats are characterized by altered explorative strategies, high risk-assessment behavior and a reactive coping style compared to SD rats. When re-tested in a familiar arena, FSL rats perform similarly to SD rats, indicating the ability to familiarize with the environment, adapt and display behavioral flexibility in agreement with a reactive coping style. In addition, in the home cage change test, FSL rat strategy of social dominance behavior relies on low aggression and low defensive behaviors, but higher avoidance. Again these behaviors are associated with a reactive coping style. Our work points to the fact that coping styles affect how animals respond to experimental testing. Thus, modeling depression in animals and translating the behavioral observations between human pathology and animal behavior would benefit from using more complex test situations allowing for the expression of a broader behavioral repertoire as well as from considering coping styles of animals.

## Author contributions

ER, SH, SM, and ML designed the experiment. SM performed the animal experiments. SH, SM, and SL analyzed the data. All authors discussed the results and contributed to the writing of the paper.

### Conflict of interest statement

The authors declare that the research was conducted in the absence of any commercial or financial relationships that could be construed as a potential conflict of interest.
