# Supplementary material for: Altered explorative strategies and reactive coping style in the FSL rat model of depression
Source: Front Behav Neurosci. 2015 Apr 21;9:89. doi: 10.3389/fnbeh.2015.00089 (PMC4404828; doi:10.3389/fnbeh.2015.00089)
Supplement: Supplementary file 1 [file Table1.PDF]

Supplementary Material: Tables

Altered explorative strategies and reactive coping style in the FSL rat model of depression

Salvatore Magara[1], Sarah Holst[1], Stina Lundberg[2], Erika Roman[2], Maria Lindskog[1]*

[1]Department of Neuroscience, Karolinska Institutet, Stockholm, Sweden
[2]Department of Pharmaceutical Biosciences, Uppsala University, Uppsala, Sweden

***Correspondence:**
Maria Lindskog
Department of Neuroscience
Karolinska Institutet
171 77 Stockholm
Sweden
Mia.Lindskog@ki.se

Number of tables: 5

Table S1
Results from the first trial (novel) of the multivariate concentric square field™ (MCSF) test in Sprague-Dawley (SD) and Flinders Sensitive Line (FSL) rats.

Functional categories	Parameters	FSL vs SD	SD			FSL		
			Median	QR	N	Median	QR	N
**General activity**	TOTAL ACT		281	183	12	270	165	12
	FRQ TOTAL CORR		81	32	12	79	39	12
	%FRQ TOTAL CORR		31.1	7.1	12	31.5	15.4	12
	FRQ CENTER		52	22	12	59	31	12
	%FRQ CENTER		18.1	9.4	12	20.1	8.5	12
	DUR CENTER		378	107	12	365	163	12
	DUR/FRQ CENTER		8.1	3.9	12	6.2	2.3	12
	DISTANCE ARENA		13537	5325	12	10719	4582	12
	DISTANCE CENTER	↓ (U=28)	4395	861	12	3601	2291	12
	VELOCITY ARENA		8	3	12	6	3	12
	VELOCITY CENTER	T	12	3	12	9	3	12
	VELOCITY TOTAL CORR	↓ (U=36)	23	9	12	17	7	12
**Exploratory activity**	LAT LEAVE		30	34	12	39	30	12
	DUR TOTAL CORR	↑ (U=33)	852	130	12	994	291	12
	DUR/FRQ TOTAL CORR		10.3	5.6	12	10.3	8.5	12
	LAT HURDLE		226.3	404.8	10	151.3	648.2	11
	FRQ HURDLE		31	13	10	23	31	11
	%FRQ HURDLE		9.7	3.2	10	9.9	7.3	11
	DUR HURDLE	T	127	92	10	81	98	11
	DUR/FRQ HURDLE	↓ (U=25)	5.2	2.6	10	3.3	2.9	11
	OCC HURDLE		10		12	11		12
	REARINGS		105	46	12	89	58	12
	NOSE POKES		6	10	10	4	9	11
**Shelter seeking**	LAT DCR		51	177	11	89	413	11
	FRQ DCR	↓ (U=24)	11	4	11	5	8	11
	%FRQ DCR	↓↓ (U=17)	4.2	2.1	11	2.0	2.1	11
	DUR DCR	T	156	119	11	74	191	11
	DUR/FRQ DCR		12.9	10.7	11	14.8	12.7	11
	OCC DCR		11		12	11		12
**Risk assessment**	LAT SLOPE		186	328	10	247	550	10
	FRQ SLOPE		19	18	10	19	23	10
	DUR SLOPE		27	30	10	44	62	10
	DUR/FRQ SLOPE		1.4	0.5	10	1.9	1.4	10
	OCC SLOPE		10		12	10		12
	LAT BE		190.3	447.9	10	248.3	553.2	10
	FRQ BE		32	19	10	38	36	10
	DUR BE		36	32	10	40	45	10
	DUR/FRQ BE		0.8	0.7	10	1	0.5	10
	OCC BE		10		12	10		12
	SAP	↑↑ (U=25)	11	11	12	24	12	12
	OCC SAP		12		12	12		12
**Risk taking**	LAT BRIDGE		191	493	10	250	556	10

	FRQ BRIDGE		41	40	10	33	30	10
	DUR BRIDGE		172	96	10	135	126	10
	DUR/FRQ BRIDGE		3.7	2.1	10	4.2	2.7	10
	OCC BRIDGE		10		12	10		12
	LAT CTRCI	↓↓ (U=15)	227	343	11	10	21	12
	FRQ CTRCI		12	16	11	14	19	12
	DUR CTRCI		9	8	11	17	20	12
	DUR/FRQ CTRCI		0.8	0.2	11	0.9	0.6	12
	OCC CTRCI		11		12	12		12
	VELOCITY CTRCI	↓↓ (U=22)	26	9	11	16	10	12
**Others**	GROOMINGS	T	6	9	12	3	4	12
	OCC GROOMING		12		12	11		12
	FECAL BOLI		0	0	12	0	0	12
	OCC BOLI		2		12	1		12
	URINE SPOTS		0	1	12	0	1	12
	OCC URINE		4		12	5		12
	BODY WEIGHT	↓↓↓ (U=0)	450	19	12	344	12	12

Table S1: Descriptive parameters recorded during the 30-min trial in the MCSF test in SD and FSL rats. Parameters are presented within the functional categories used for interpretation. The table reports median, interquartile range (QR) and number of rats (N).

↑ p<0.05, ↑↑ p<0.01, ↑↑↑ p<0.001 (FSL > SD, Mann-Whitney U-test). ↓ p<0.05, ↓↓ p<0.01, ↓↓↓ p<0.001 (FSL < SD, Mann-Whitney U-test). T = trend (0.05<p≤0.1, Mann-Whitney U-test).

Table S2

Results from the second trial (familiar) of the multivariate concentric square field™ (MCSF) test in Sprague-Dawley (SD) and Flinders Sensitive Line (FSL) rats.

Functional categories	Parameters	FSL vs SD	SD			FSL		
			Median	QR	N	Median	QR	N
**General activity**	TOTAL ACT		225	61	12	230	110	12
	FRQ TOTAL CORR		65	22	12	63	21	12
	%FRQ TOTAL CORR	T	29.2	3.6	12	25.3	11.4	12
	FRQ CENTER		63	28	12	56	24	12
	%FRQ CENTER		25.8	11.7	12	21.9	8.6	12
	DUR CENTER	T	340	193	12	284	95	12
	DUR/FRQ CENTER		6.5	2.5	12	5.4	2.4	12
	DISTANCE ARENA		11900	3800	12	11060	4512	12
	DISTANCE CENTER	↓ (U=37)	4105	994	12	2848	1935	12
	VELOCITY ARENA		7	2	12	6	2	12
	VELOCITY CENTER		11	4	12	11	3	12
	VELOCITY TOTAL CORR		19	6	12	20	6	12
**Exploratory activity**	LAT LEAVE		6	5	12	9	19	12
	DUR TOTAL CORR		884	221	12	815	227	12
	DUR/FRQ TOTAL CORR		12.6	7.2	12	13.2	4.9	12
	LAT HURDLE		72.6	232.3	11	151.5	147.9	11
	FRQ HURDLE		16	9	11	18	16	11
	%FRQ HURDLE		8.1	3.0	11	6.0	5.2	11
	DUR HURDLE		141	85	11	176	126	11
	DUR/FRQ HURDLE		8.9	6.4	11	10.2	6.1	11
	OCC HURDLE		10		12	11		12
	REARINGS		90	32	12	111	48	12
	NOSE POKES		6	7	11	11	18	11
**Shelter seeking**	LAT DCR	↑ (U=34)	36	135	12	213	289	12
	FRQ DCR	↓ (U=34)	11	4	12	9	7	12
	%FRQ DCR	↓ (U=33)	5.4	2.7	12	3.9	3.2	12
	DUR DCR		132	220	12	142	104	12
	DUR/FRQ DCR		14	13	12	17	21.8	12
	OCC DCR		12		12	12		12
**Risk assessment**	LAT SLOPE		259	147	11	303	262	11
	FRQ SLOPE		14	10	11	30	30	11
	DUR SLOPE		28	22	11	57	50	11
	DUR/FRQ SLOPE		2	1.1	11	1.8	1.6	11
	OCC SLOPE		11		12	11		12
	LAT BE		274.2	269.8	11	399.0	322.2	11
	FRQ BE	T	17	14	11	35	29	11
	DUR BE	↑ (U=30)	21	22	11	48	58	11
	DUR/FRQ BE		1.1	0.8	11	1.7	1.6	11
	OCC BE		11		12	11		12
	SAP		9	14	12	7	6	12
	OCC SAP		11		12	11		12
**Risk taking**	LAT BRIDGE		276	270	11	400	317	11

	FRQ BRIDGE		23	18	11	29	21	11
	DUR BRIDGE		135	85	11	193	122	11
	DUR/FRQ BRIDGE		5.8	5.5	11	6.8	2.1	11
	OCC BRIDGE		11		12	11		12
	LAT CTRCI		98	230	12	66	161	10
	FRQ CTRCI		14	11	12	20	18	10
	DUR CTRCI		10	5	12	14	20	10
	DUR/FRQ CTRCI		0.8	0.6	12	1	0.6	10
	OCC CTRCI		12		12	10		12
	VELOCITY CTRCI		24	21	12	20	15	10
**Others**	GROOMINGS	↓↓ (U=21)	10	12	12	1	2	12
	OCC GROOMING		11		12	9		12
	FECAL BOLI		0	0	12	0	0	12
	OCC BOLI		0		12	0		12
	URINE SPOTS	T	0	1	12	0	0	12
	OCC URINE		3		12	0		12
	BODY WEIGHT	↓↓↓ (U=0)	525	60	12	390	22	12

Table S2: Descriptive parameters recorded during the 30-min trial in the second MCSF test in SD and FSL rats. Parameters are presented within the functional categories used for interpretation. The table reports median, interquartile range (QR) and number of rats (N).
↑ $p<0.05$, ↑↑ $p<0.01$, ↑↑↑ $p<0.001$ (FSL > SD, Mann-Whitney U-test). ↓ $p<0.05$, ↓↓ $p<0.01$, ↓↓↓ $p<0.001$ (FSL < SD, Mann-Whitney U-test). T = trend ($0.05<p\leq0.1$, Mann-Whitney U-test).

Table S3
Differences between the first (novel) and the second (familiar) trial of the multivariate concentric square field™ (MCSF) test in Flinders Sensitive Line (FSL) and Sprague-Dawley (SD) rats.

Functional categories	Parameters	FSL	SD
**General activity**	TOTAL ACT		T
	FRQ TOTAL CORR	↓↓	↓
	%FRQ TOTAL CORR		
	FRQ CENTER		
	%FRQ CENTER		↑
	DUR CENTER	↓	
	DUR/FRQ CENTER	T	T
	DISTANCE ARENA		T
	DISTANCE CENTER		
	VELOCITY ARENA		T
	VELOCITY CENTER	T	
	VELOCITY TOTAL CORR	T	↓
**Exploratory activity**	LAT LEAVE	↓↓↓	↓↓
	DUR TOTAL CORR	↓	
	DUR/FRQ TOTAL CORR		↑
	LAT HURDLE		↓
	FRQ HURDLE		↓
	%FRQ HURDLE		
	DUR HURDLE	↑	
	DUR/FRQ HURDLE	↑↑↑	↑↑
	OCC HURDLE		
	REARINGS		
	NOSE POKES	↑	
**Shelter seeking**	LAT DCR		↓
	FRQ DCR		
	%FRQ DCR	T	
	DUR DCR		
	DUR/FRQ DCR		
	OCC DCR		
**Risk assessment**	LAT SLOPE		
	FRQ SLOPE		
	DUR SLOPE		
	DUR/FRQ SLOPE		
	OCC SLOPE		
	LAT BE		
	FRQ BE		
	DUR BE		
	DUR/FRQ BE		
	OCC BE		
	SAP	↓↓↓	
	OCC SAP		
**Risk taking**	LAT BRIDGE		
	FRQ BRIDGE		↓

	DUR BRIDGE		
	DUR/FRQ BRIDGE	T	↑
	OCC BRIDGE		
	LAT CTRCI	T	T
	FRQ CTRCI		
	DUR CTRCI		
	DUR/FRQ CTRCI		
	OCC CTRCI		
	VELOCITY CTRCI		
**Others**	GROOMINGS		T
	OCC GROOMING		
	FECAL BOLI		
	OCC BOLI		
	URINE SPOTS		
	OCC URINE		
	BODY WEIGHT	↑↑	↑↑↑

Table S3: Within-group comparison of performance in trial 2 versus trial 1 in the MCSF test. ↑ $p<0.05$, ↑↑ $p<0.01$, ↑↑↑ $p<0.001$ (trial 2 > trial 1, Wilcoxon matched pairs test). ↓ $p<0.05$, ↓↓ $p<0.01$, ↓↓↓ $p<0.001$ (trial 2 < trial 1, Wilcoxon matched pairs test). T = trend ($0.05<p\leq0.1$, Wilcoxon matched pairs test).

Table S4

Results from the novel cage test in Sprague-Dawley (SD) and Flinders Sensitive Line (FSL) rats.

Functional categories	Parameters	FSL vs SD	SD			FSL		
			Median	QR	N	Median	QR	N
**Proactive coping**	DUR ST. APPROACH		0.08	0.15	12	0.12	0.03	12
	DUR SAP		0.03	0.02	12	0.04	0.03	12
	DUR GROOMING		0.06	0.04	12	0.04	0.04	12
	FRQ ST. APPROACH		0.11	0.26	12	0.18	0.04	12
	FRQ SAP		0.07	0.04	12	0.07	0.06	12
	FRQ GROOMING		0.01	0.02	12	0.02	0.01	12
	OCC ST. APPROACH		12		12	12		12
	OCC SAP		12		12	12		12
	OCC GROOMING		10		12	12		12
**Reactive coping**	DUR FREEZING		0.04	0.03	12	0.05	0.07	12
	DUR MOTIONLESS	↑↑ (U=23)	0.04	0.05	12	0.11	0.04	12
	FRQ FREEZING		0.04	0.03	12	0.04	0.04	12
	FRQ MOTIONLESS	↑ (U=27)	0.04	0.04	12	0.09	0.04	12
	OCC FREEZING		12		12	11		12
	OCC MOTIONLESS		12		12	12		12
**Exploratory activity**	DUR FREE REARING		0.04	0.05	12	0.06	0.08	12
	DUR INVEST.	↓↓ (U=20)	0.41	0.07	12	0.31	0.07	12
	FRQ FREE REARING		0.05	0.03	12	0.06	0.07	12
	FRQ INVEST.	↓ (U=32)	0.35	0.06	12	0.31	0.07	12
	OCC FREE REARING		12		12	12		12
	OCC INVEST.		12		12	12		12
**Locomotor activity**	DUR WALL REARING		0.16	0.06	12	0.17	0.05	12
	DUR WALKING		0.09	0.11	12	0.08	0.08	12
	FRQ WALL REARING		0.12	0.04	12	0.12	0.03	12
	FRQ WALKING		0.18	0.21	12	0.11	0.09	12
	OCC WALL REARING		12		12	12		12
	OCC WALKING		11		12	11		12

Table S4: Individual behaviors scored in the novel cage test in SD and FSL rats. Parameters are presented within the functional categories used for interpretation of coping styles. Duration (DUR), frequency (FRQ) and occurrence (OCC) of the individual behaviors (described in Table 2) are expressed as fraction of the total behavior scored. The table reports median, interquartile range (QR) and number of rats (N). Abbreviations: ST. APPROACH=stretched approach, SAP=stretched attend postures, INVEST=investigating.

↑ $p<0.05$, ↑↑ $p<0.01$, ↑↑↑ $p<0.001$ (FSL > SD, Mann-Whitney U-test). ↓ $p<0.05$, ↓↓ $p<0.01$, ↓↓↓ $p<0.001$ (FSL < SD, Mann-Whitney U-test).

Table S5
Results from the home cage change test in Sprague-Dawley (SD) and Flinders Sensitive Line (FSL) rats.

Functional categories	Parameters	FSL vs SD	SD			FSL		
			Median	QR	N	Median	QR	N
**Neutral behavior**	DUR HEAD-HEAD		0.08	0.08	12	0.07	0.06	12
	DUR NOSE-SIDE		0.11	0.02	12	0.12	0.06	12
	DUR NOSE-NOSE		0.02	0.01	12	0.01	0.02	12
	DUR PASSING		0.11	0.05	12	0.13	0.05	12
	FRQ HEAD-HEAD	T	0.09	0.05	12	0.07	0.02	12
	FRQ NOSE-SIDE		0.14	0.03	12	0.15	0.06	12
	FRQ NOSE-NOSE	↓ (U=37)	0.02	0.02	12	0.01	0.01	12
	FRQ PASSING		0.15	0.05	12	0.14	0.05	12
	OCC HEAD-HEAD		12		12	12		12
	OCC NOSE-SIDE		12		12	12		12
	OCC NOSE-NOSE		12		12	10		12
	OCC PASSING		12		12	12		12
**Dominant behavior**	DUR HEAD-TAIL		0.04	0.02	12	0.06	0.04	12
	DUR NOSE-GENITALS		0.03	0.03	12	0.04	0.07	12
	DUR FOLLOWING		0.06	0.06	12	0.07	0.02	12
	DUR APPROACHING		0.06	0.04	12	0.08	0.04	12
	DUR NUZZLING	↓ (U=34)	0.11	0.09	12	0.06	0.09	12
	DUR MOUNT 1	↑ (U=31)	0.03	0.02	12	0.06	0.03	12
	FRQ HEAD-TAIL	T	0.04	0.02	12	0.06	0.02	12
	FRQ NOSE-GENITALS	T	0.03	0.04	12	0.05	0.07	12
	FRQ FOLLOWING		0.06	0.06	12	0.08	0.05	12
	FRQ APPROACHING		0.07	0.03	12	0.08	0.02	12
	FRQ NUZZLING	↓↓ (U=21)	0.11	0.05	12	0.07	0.06	12
	FRQ MOUNT 1	↑↑ (U=25)	0.02	0.02	12	0.05	0.02	12
	OCC HEAD-TAIL		12		12	12		12
	OCC NOSE-GENITALS		12		12	12		12
	OCC FOLLOWING		11		12	12		12
	OCC APPROACHING		12		12	12		12
	OCC NUZZLING		12		12	12		12
	OCC MOUNT 1		12		12	12		12
**Aggressive behavior**	DUR MOUNT 2		0.00	0.01	12	0.00	0.00	12
	DUR CHASING		0.00	0.01	12	0.00	0.01	12
	DUR FIGHT	↓↓↓ (U=10)	0.09	0.09	12	0.00	0.03	12
	FRQ MOUNT 2		0.00	0.01	12	0.00	0.00	12
	FRQ CHASING		0.00	0.01	12	0.00	0.02	12
	FRQ FIGHT	↓↓↓ (U=7)	0.05	0.04	12	0.00	0.02	12
	OCC MOUNT 2		4		12	3		12
	OCC CHASING		5		12	4		12
	OCC FIGHT	↓ (p=0.0137)	12		12	6		12
**Submissive behavior**	DUR AVOIDING	↑↑ (U=19)	0.09	0.05	12	0.17	0.10	12
	DUR CROWING		0.01	0.03	12	0.02	0.02	12
	DUR SUBMISS. POST.	↓↓↓ (U=17)	0.05	0.07	12	0.00	0.00	12
	FRQ AVOIDING	↑↑ (U=27)	0.11	0.07	12	0.17	0.07	12
	FRQ CROWING		0.01	0.03	12	0.02	0.02	12
	FRQ SUBMISS. POST.	↓↓↓ (U=15)	0.02	0.02	12	0.00	0.00	12

OCC AVOIDING		12	12	12	12
OCC CROWING		10	12	11	12
OCC SUBMISS. POST.	↓ ($p=0.0123$)	10	12	3	12

Table S5: Social behaviors scored in the home cage change test in SD and FSL rats. Parameters are presented within the functional categories used for interpretation of social behaviors. Duration (DUR), frequency (FRQ) and occurrence (OCC) of the single social behaviors (described in Table 2) are expressed as fraction of the total behavior scored. The table reports median, interquartile range (QR) and number of rats (N). Abbreviations: SUBMISS. POST.=submissive posture.
↑ $p<0.05$, ↑↑ $p<0.01$, ↑↑↑ $p<0.001$ (FSL > SD, Mann-Whitney U-test). ↓ $p<0.05$, ↓↓ $p<0.01$, ↓↓↓ $p<0.001$ (FSL < SD, Mann-Whitney U-test). T = trend ($0.05<p\leq0.1$, Mann-Whitney U-test).
