# Supplementary material for: Altered explorative strategies and reactive coping style in the FSL rat model of depression
Source: Front Behav Neurosci. 2015 Apr 21;9:89. doi: 10.3389/fnbeh.2015.00089 (PMC4404828; doi:10.3389/fnbeh.2015.00089)
Supplement: Supplementary file 3 [file Image1.PDF]

Supplementary Material: Figures

Altered explorative strategies and reactive coping style in the FSL rat model of depression

Salvatore Magara[1], Sarah Holst[1], Stina Lundberg[2], Erika Roman[2], Maria Lindskog[1]*

[1]Department of Neuroscience, Karolinska Institutet, Stockholm, Sweden
[2]Department of Pharmaceutical Biosciences, Uppsala University, Uppsala, Sweden

***Correspondence:**
Maria Lindskog
Department of Neuroscience
Karolinska Institutet
171 77 Stockholm
Sweden
Mia.Lindskog@ki.se

Number of figures: 2

Figure S1

The partial least squares discriminant analysis (PLS-DA) of data from the first MCSF test.

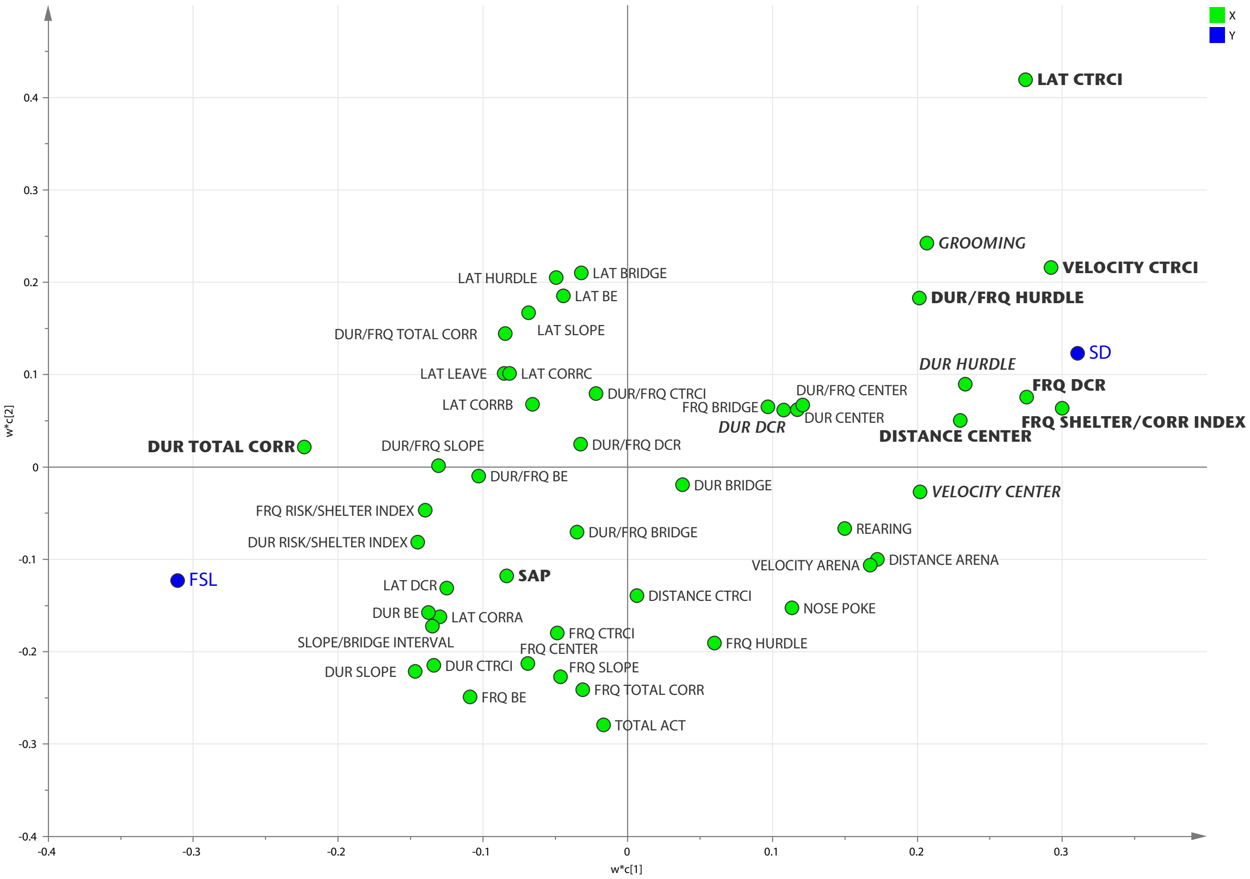

Figure S1: The PLS-DA loading plot showing how the descriptive parameters (green dots) from the first (novel) MCSF test load in relation to the experimental groups (blue dots), i.e. SD and FSL. PLS-DA scatter plot of the two first components (R^2X (cum)=0.348, R^2Y (cum)=0.748, Q^2 (cum)=0.279, first component significant). For clarity, parameters that are significantly different between groups as well as display a trend towards significance using the Mann-Whitney U-test (Table S1) are indicated in bold and italics, respectively. Abbreviations are reported in Table 1.

Figure S2
Principal component analysis (PCA) of data from the novel cage test.

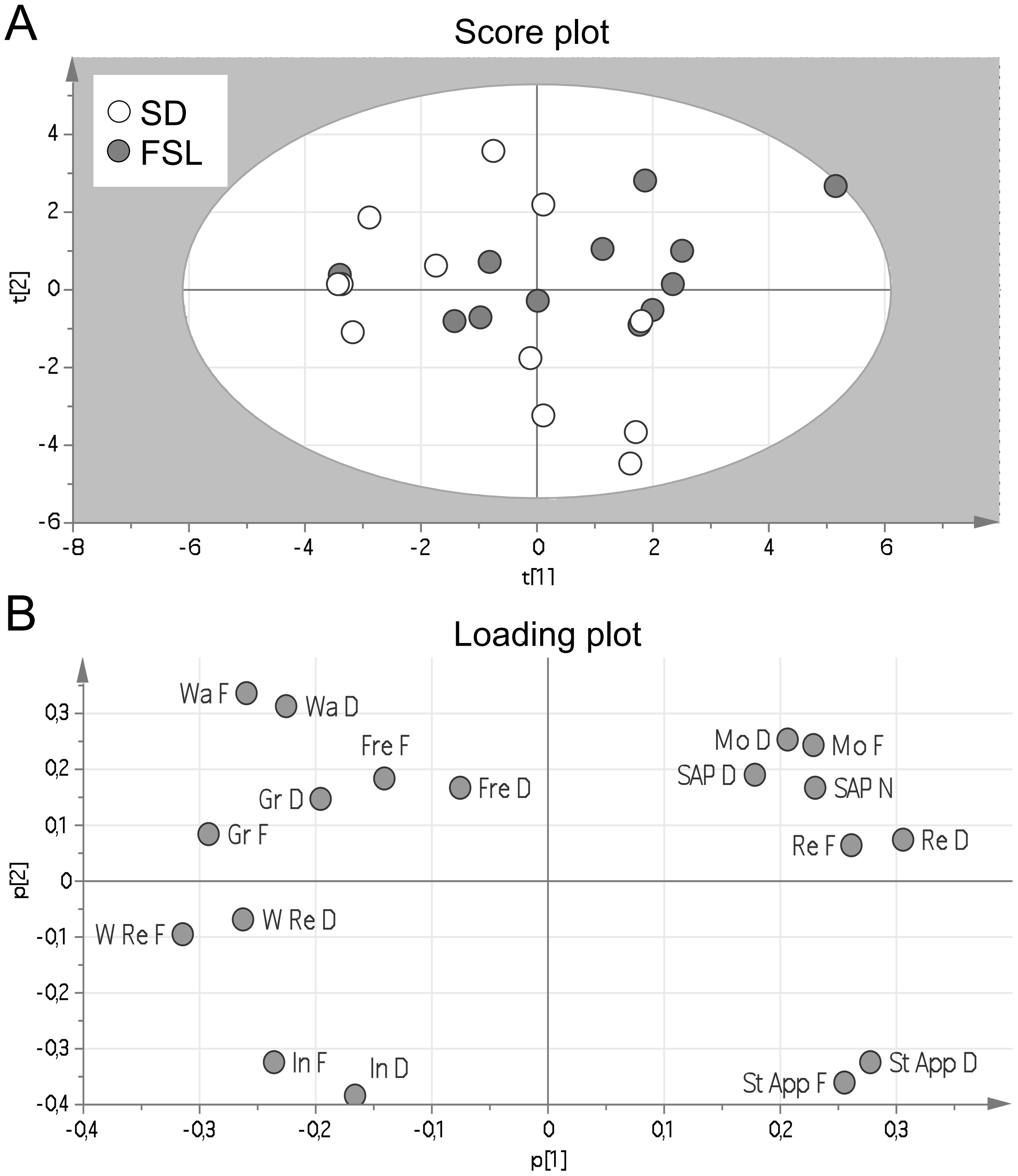

Figure S1: Principal component analysis score plot (A) showing individual SD and FSL rats, and loading plot (B) of individual behaviors in the novel cage test. Abbreviations: Fre=Freezing, Gr=Grooming, In=Investigating, Mo=Motionless, Re=Rearing, W Re=Wall Rearing, St App=Stretched Approach, SAP=Stretched Attend Posture, Wa=Walking, D=Duration of the behavior, F=frequency of the behavior. Duration and frequency were calculated as fraction of the total behavior scored per rat.
